# Annotations

**Published:** 1895-08-10

**Authors:** 


### ANNOTATIONS.

### The Morals of a Man of Science.

What a man does is the proof to the world of what a man is. Many good people fear that the advance of science will bring about the retrogression of morals and religion. We do not agree with them. But if they cannot accept our judgment let them weigh well a fact like this. Mr. Jonathan Hutchinson, F.R.S., an ex-president of the Royal College of Surgeons, addressed his professional brethren assembled in annual congress the other day, and he thus spoke: "I bore with such equanimity as I could the discovery that I could not compete with my friend in the ratio of successes obtained" (in operations for ovariotomy), "and, acting on the rule of conduct that I would never keep a patient in my own hands if I believed that someone else could do what was needed with greater prospect of success, I gave up doing ovariotomies, both in public and in private, and used to transfer my patients from the London to the Samaritan Hospital." Here is a rule of conduct which has never been excelled in moral worth in any department of professional life or private behaviour. What would be the effect if, for example, every lawyer should insist upon transferring clients who might need the services of others more specially experienced and competent than himself, or if every tradesman should send customers to other tradesmen who might happen to be able to supply certain classes of goods better and more cheaply than himself? Nay, may we not ask what the effect would be if every clergyman were to send certain classes of his hearers to other clergymen who might be more specially learned and competent in certain particulars than himself? A most far-reaching and truly noble rule is this of Mr. Jonathan Hutchinson's; and the fact that he announced it towards the close of his career in the hearing of hundreds of his professional brethren, who are almost as familiar as he is himself with the conduct of his professional life, is proof that he spoke mere truth. If these are the morals of men of science, may we not say of men of all professions and callings, *O si sic omnes!*

### The Doctor and the Lawyer: An Ancient Conflict.

The writer has in his possession an old print representing a patient in the agonies of death, with his wife and helpless children weeping round the bed, and the doctor and family attorney in a corner of the room engaged in a physical conflict so desperate that it would appear as if it could only terminate with the death of one or other of them. The strife which used to rage so hotly round the bed of the dying is now waged with hardly less fury round the body of the insane. Dr. Maudsley very ably represents the medical profession in this sturdy, but soon-to-be-ended conflict. In the section of psychology at the annual meeting of the British Medical Association the other day, he reviewed the old arguments in favour of the scientific medical specialists' views of criminal responsibility, and the crude, antiquated, and unscientific position taken up by lawyers and judges. Dr. Maudsley claims that medical science has succeeded in winning the community to its side, and he thence concludes that the conversion of the lawyers is a mere question of time. But, as a matter of fact, the lawyers have not a leg to stand upon. There is only one point really at issue, and it is this: whether is likely to be the better judge of criminal responsibility in a person, the educated man who is specially trained and devotes his whole life to the study of every form of human responsibility, or the man who is not trained, and who does not devote himself to such study at all, except accidentally, and on occasions which are comparatively rare? To state the problem is to solve it. In this case the doctor is the expert, and the lawyer is not; and the untrained must go down before the specialist. It is a pity that the lawyers continue to maintain so unscientific and unworthy a fight on the subject. If they would but adopt Mr. Jonathan Hutchinson's rule, referred to in another annotation, and refuse to keep any case in their hands which could be more competently dealt with by persons other than themselves, they would confer a great advantage upon the community, and display a much more adequate judgment of what culture and professional honour demand.

### The Throat Hospital, Golden Square.

The curt and unexplained announcement that the Duke of Sutherland, lately President; Mr. R. Courtenay Welch, lately a member, and for nearly twelve years Chairman of the Committee of Management; and Dr. Greville MacDonald, lately Dean of the Medical School, and for eight years Physician to the Hospital, have entirely severed their connection with the Throat Hospital in Golden Square, suggests that, notwithstanding the peace which has been supposed to reign during recent years, all has not been harmony in that once turbulent institution. We cannot but regret, however, that the three gentlemen who, by a letter to the press, have so publicly announced their retirement from the hospital, should have thought fit to withhold their reasons for so doing. The public are but slightly interested in the doings of any of these gentlemen, and as the only thing which gives any importance to their action is that it imputes blame to the management of the hospital, it would have been well if they had stated in an equally public manner the nature of the evils against which they intend it as a protest. If it is true that an attempt has been made to force upon the committee of management a small band hostile to the best interests of the hospital, surely it would have been better to say so, and to warn the public as to what was going on; if on the other hand this intrusion of new blood was made with the object of breaking up a clique, then the managers should, for the credit of their hospital, lay before the public the true state of affairs. We are quite aware of the unpleasantness which took place at the annual meeting, of all the rumours current regarding the legality or otherwise of the proceedings, and of the events which have since then developed in the conduct of the committee; but what we wish specially to insist upon is that the gentlemen who have so advertised their retirement should state their reasons, and on the other hand that the managers of an institution, on which such a slur has been cast, should explain the position. If neither of these courses is adopted, the subscribers should demand a complete investigation. No hospital can carry out its functions properly unless those by whom it is managed act harmoniously together with that object, and any intrusion of private aims or of private animosities should be promptly suppressed by the subscribing public.

